# Plant defense compounds can enhance antagonistic effects against *Alternaria brassicicola* of seed-associated fungi isolated from wild Brassicaceae

**DOI:** 10.3389/fpls.2024.1466043

**Published:** 2024-09-30

**Authors:** Thomas Lerenard, Sophie Aligon, Romain Berruyer, Pascal Poupard, Josiane Le Corff

**Affiliations:** Université d’Angers, INRAe, Institut Agro, UMR 1345 IRHS, SFR 4207 QUASAV, Angers, France

**Keywords:** *Alternaria brassicicola*, glucosinolates, camalexin, antagonistic fungi, synergy

## Abstract

Plant microbiota appear more and more as potential sources of antagonistic microorganisms. However, the seed microbiota associated with wild plant species has rarely been explored. To identify fungal antagonists to the seed-borne pathogen *Alternaria brassicicola*, seeds were collected in natural populations of three Brassicaceae species, *Arabidopsis thaliana*, *Capsella bursa-pastoris* and *Draba verna*. A large number of fungal strains reduced the growth of *A. brassicicola*. The most antagonistic strains belonged to *Alternaria*, *Apiospora*, *Trichoderma* and *Aspergillus*. Seed-associated fungi tolerated host plant defenses and exhibited lower sensitivity compared to *A. brassicicola* to indolic compounds such as the phytoalexin camalexin and the glucosinolates (GLS)-breakdown compound indole-3-carbinol. By contrast, antagonistic strains were as inhibited as *A. brassicicola* in presence of allyl-isothiocyanates (ITC) derived from aliphatic GLS, and more inhibited by benzyl-ITC derived from aromatic GLS. However, all defense compounds could enhance the antagonistic effects of some of the isolated strains on *A. brassicicola*. The observed potential synergistic effects between defense compounds and seed-associated antagonistic strains emphasize the need for further studies to elucidate the molecular bases of the interactions. A better understanding of the interactions between host plants, pathogens and fungal endophytes is also needed to develop sustainable biocontrol strategies.

## 1 Introduction

Plant diseases cause considerable yield losses and represent a major threat for most crops (Rashad and Moussa, 2020). To minimize their negative impacts, for more than 50 years, the use of conventional fungicides has been the main management approach with consequences such as selection of resistant strains of pathogens and major environmental and health-related problems (Corkley et al., 2022). As another consequence, in recent years, implementing safe management measures has been under steady research development. In this context, plant microbiota appear more and more as potential sources of microorganisms that benefit their hosts through protection against diseases, reduction or suppression of pathogen growth and reproduction (Berg et al., 2017; Trivedi et al., 2020; Solanki et al., 2021). However, while in the leaf and root microbiota, a number of biocontrol agents (BCA) have already been identified to manage plant pathogens (Vionnet et al., 2018; Xie et al., 2020; Cobos et al., 2022; Correia et al., 2022), seed microbiota have been largely unexplored (Berg et al., 2017).

The seed-borne necrotrophic pathogen *Alternaria brassicicola* causes significant damage in terms of seed quality, germination and crop yields (Chirco and Harman, 1978; Pattanamahakul and Strange, 1999). This fungus, responsible of black spot disease and damping-off in *Brassica* crops, can be transmitted to the seeds which represent the primary source of inoculum (Pochon et al., 2012; Guillemette et al., 2014). To date, chemical control is the main management method (Saharan et al., 2016), and better understanding of resistance mechanisms in Brassicaceae plants is needed to develop sustainable disease control strategies. Intriguingly, some wild species in the Brassicaceae family have been described as resistant to *A. brassicicola* (Cooke et al., 1997; Westman et al., 1999). As wild plant species can harbor antagonists to plant pathogens (Luu et al., 2021; Ilic, 2023), their role as a source of novel BCA should be investigated and, in particular, whether resistant wild Brassicaceae species host in their seed microbiota antagonistic fungi with an inhibitory effect on *A. brassicicola*.

During infection of Brassicaceae species, *A. brassicicola* kills plant cells and grows on their contents while being exposed to high levels of antimicrobial compounds (Tao et al., 2022). These metabolites can be either induced by pathogen attacks such as phytoalexins, or constitutive such as phytoanticipins. In the absence of the indolic phytoalexin camalexin, enhanced susceptibility to *A. brassicicola* is observed (Thomma et al., 1999; Tao et al., 2022). Moreover, upon tissue damage, hydrolysis of the phytoanticipins glucosinolates (GLS) by myrosinases yields isothiocyanates, nitriles and epithionitriles (Halkier and Gershenzon, 2006). Isothiocyanates (ITC), the most common breakdown products, exhibit toxicity towards several plant pathogens, including *A. brassicicola* (reviewed in Plaszkó et al., 2021). In particular, *in vitro* studies demonstrate the inhibitory effects of ITC derived from the three glucosinolate classes synthesized by wild Brassicaceae (aliphatic, aromatic and indolic; Fahey et al., 2001) on *A. brassicicola* (Sellam et al., 2007b; Pedras and Hossain, 2011; Calmes et al., 2015). Nonetheless, inhibitory effects do differ among GLS classes and especially among their degradation products (Sellam et al., 2007b; Joubert et al., 2011; Buxdorf et al., 2013; Calmes et al., 2015).

Similarly, potential antagonistic fungi inhabiting the seed microbiota, are also exposed to plant secondary compounds (Latz et al., 2018). However, to our knowledge, no study has investigated the sensitivity of non-pathogenic seed-associated fungi to plant defenses, and more generally only a few studies have examined the effects of plant metabolites on endophytic fungi (Poveda et al., 2022). One example is given by Szűcs et al. (2018) who identified fungal endophytes from roots of the horseradish *Armoracia rusticana*. Their results show that the majority of the isolated strains were not inhibited by GLS encountered in the host plant as most of them could decompose aliphatic and aromatic GLS into ITC. Some endophytes could also use one aliphatic GLS as source of carbon. As a consequence, higher potential tolerance to the plant defense compounds combined with the use of the host GLS as nutrients could give endophytes a competitive advantage over other species that are more impacted by the plant defense compounds, and in particular pathogens. However, this hypothesis was not addressed by Szűcs et al. (2018). Furthermore, antagonistic fungal endophytes may suppress plant pathogens by direct inhibition through antibiosis, competition or mycoparasitism, or indirect inhibition through induction of plant defenses (Latz et al., 2018). Often, several mechanisms are active at the same time, but direct inhibition through antibiosis mediated by toxic metabolites seems quite common (Card et al., 2015). As synergistic activity between ITC and non-ITC antifungal agents against a few pathogens has been reported (reviewed in Plaszkó et al., 2021), combination of plant defense compounds and fungal metabolites could potentially enhance antagonistic effects of fungi isolated from wild Brassicaceae.

The aim of the current study was to get insights into the role of wild Brassicaceae defense compounds on the interaction between *A. brassicicola* and potential antagonistic fungi. The main questions were: 1) Do fungal strains isolated from wild Brassicaceae seeds have an inhibitory effect on *A. brassicicola*? 2) Are there differences in sensitivity to the plant defense compounds between *A. brassicicola* and antagonistic strains? and 3) Could host defense compounds reinforce the antagonistic impact of seed-associated fungi on *A. brassicicola*? To answer these questions, we first isolated seed-associated fungi from different wild Brassicaceae species and conducted dual cultures assays on solid medium to identify and select the strains with the highest antagonistic potential. *Alternaria brassicicola* growth inhibition by culture filtrates from the selected strains was then measured in liquid media using nephelometric assays. Secondly, to assess the impacts of defense compounds on *A. brassicicola* and on the isolated antagonistic fungi, we measured their growth in liquid media amended with defense compounds synthesized by the wild Brassicaceae species in our sample. Finally, the growth of *A. brassicicola* was measured in liquid media amended with defense compounds and antagonistic culture filtrates, and we tested their potential synergistic effects on the pathogen.

## 2 Material and methods

### 2.1 Isolation of fungal strains from wild Brassicaceae seeds

In April 2022, seeds from three species of wild Brassicaceae were collected in three sites with contrasted environmental conditions around Angers, France (**Table 1**). The three plant species were *Arabidopsis thaliana* (L.) Heynh., *Capsella bursa-pastoris* (L.) Medik and *Draba verna* L., characterized by different metabolite profiles: all three species synthesize aliphatic GLS while camalexin and indolic GLS are only present in *A. thaliana* and *C. bursa-pastoris*, and aromatic GLS in *A. thaliana* and *D. verna* (Daxenbichler et al., 1991; Cole, 1976; Kagan and Hammerschmidt, 2002; Jimenez et al., 1997; Czerniawski et al., 2021; Bréard et al., 2022). *Arabidopsis thaliana* and *C. bursa-pastoris* have been described as resistant to *A. brassicicola* (Cooke et al., 1997; Westman et al., 1999), there is no data on *D. verna*. Within 24 h after collection, and removal of all plant residues, 100 seeds from each of the nine plant populations were evenly placed in 20 Petri dishes (90-mm diameter). Ten Petri dishes contained a Potato Dextrose Agar (PDA 39 g/L, BIOKAR Diagnostics) amended with streptomycin (500 mg/L, Sigma-Aldrich) and the remaining 10 dishes, a semi-selective medium adapted from Dhingra and Sinclair (1985) with PDA (39 g/L) amended with 150 mg/L of Rose Bengal and 250 mg/L of chloramphenicol (Sigma-Aldrich). Plates were incubated at 22°C in the dark and monitored daily. In the presence of fungal colonies, explants from the periphery of each colony were transplanted onto PDA medium and incubated in a dark environment at 22°C for 7-15 days, depending on the growth rate. This step was repeated to obtain monocultures. For long-term storage, 5-mm cylindrical mycelial explants from the periphery of each fungal colony were transferred to cryotubes containing 30% sterile glycerol in ultrapure water (v/v) and stored at -80°C. For all subsequent assays, when needed, mycelial explants were transferred to Petri dishes with PDA to obtain fresh cultures.

**Table 1:** Origin of the antagonistic strains isolated from wild Brassicaceae seeds, species identification, and GenBank accession numbers of the *ITS, tef1-α, rpb2, TUB* and *LSU* sequences.

Isolate reference	Plant species	GPS coordinates of the sampling site	*ITS* Accession number	*tef1-α* Accession number	*rpb2* Accession number	*TUB* Accession number	*LSU* Accession number	Species identification	Inhibition of *A. brassicicola* (% ± S.D.)
At 199	*A. thaliana*	47°25’38.0”N 0°31’48.3”W	PP159085	PP179971	NA	PP179968	PP237267	*Apiospora kogelbergensis*	51.9 ± 1.6
At 289	*A. thaliana*	47°25’38.0”N 0°31’48.3”W	PP159086	PP179972	PP179978	NA	NA	*Alternaria arborescens*	43.5 ± 1.9
At 416	*A. thaliana*	47°25’38.0”N 0°31’48.3”W	PP159089	PP179975	PP179980	NA	NA	*Alternaria arborescens*	46.1 ± 0.5
Cb 322	*C. bursa-pastoris*	47°28’51.0”N 0°36’30.7”W	PP159087	PP179973	NA	PP179969	PP237268	*Apiospora arundinis*	61.7 ± 3.2
Cb 324	*C. bursa-pastoris*	47°28’51.0”N 0°36’30.7”W	PP159088	PP179974	PP179979	NA	NA	*Alternaria arborescens*	38.3 ± 3.2
Cb 087	*C. bursa-pastoris*	47°25’18.3”N 0°34’42.4”W	PP159084	PP179970	PP179977	NA	NA	*Trichoderma viridarium*	52.9 ± 2.5
Dv 245	*D. verna*	47°28’51.0”N 0°36’30.7”W	PP159090	PP179976	PP179981	NA	NA	*Alternaria arborescens*	28.7 ± 5.9
Dv 028	*D. verna*	47°25’34.9”N 0°33’54.0”W	PP159091	NA	NA	NA	NA	*Aspergillus niger*	41.8 ± 2.0
Dv 101	*D. verna*	47°25’34.9”N 0°33’54.0”W	PP159092	NA	NA	NA	NA	*Aspergillus niger*	39.4 ± 3.7

Isolate references give the plant species (At, *A. thaliana*; Cb, *C. bursa-pastoris* and Dv, *D. verna*) and the individual strain number. NA indicates that the molecular markers were not used for a given isolate. For each host plant and independently of sampling sites, the three sporulating strains with the highest antagonistic potential against *A. brassicicola* on solid PDA medium were selected. Percent inhibition (± S.D.) of *A. brassicicola* is indicated. Each test was repeated three times.

### 2.2 *In vitro* screening to identify antagonistic strains against *A. brassicicola* on solid medium

All isolated strains were tested as potential antagonists against *A. brassicicola* by dual culture assays. The fungal pathogen under investigation was *A. brassicicola* strain Abra43, isolated from radish seeds in 1999 in France (Iacomi-Vasilescu et al., 2004), and routinely grown and maintained on PDA in our collection. Two 5-mm diameter mycelial discs were taken from 7-day-old PDA culture plates, one disc from a *A. brassicicola* colony and one from a fungal colony isolated from the Brassicaceae seed populations, then placed at opposite edges of 90-mm diameter Petri dishes containing sterile PDA medium. As control, a single *A. brassicicola* disc was placed at the edge of a Petri dish. Each test was repeated three times. Plates were incubated in the dark at 22°C for 10 days. At three, six, eight and 10 days, a line at the periphery of each colony was drawn on the underside of the Petri dishes. At the end of the incubation period, radial growth of the *A. brassicicola* colonies was then estimated in presence or not of the tested fungal strains.

### 2.3 Identification of the antagonistic fungal strains: DNA extraction, PCR amplification and sequencing

Among all tested strains, nine sporulating isolates with the highest inhibition activity towards of *A. brassicicola* were selected. For identification of these antagonistic strains, DNA extraction was conducted using the Nucleospin96 for food kit (Macherey-Nagel). Universal primers *ITS1* and *ITS4* were used for amplification of the *ITS* region of the rDNA (White et al., 1990). PCR reaction adapted from Guillemette et al. (2004) was performed in a total volume of 25 μL with 10µL of undiluted DNA instead of 2.5 µL. The PCR cycling protocol consisted of an initial denaturation at 95°C for 3 min, 35 cycles of denaturation at 95°C for 30s, annealing at 54°C for 50 s and elongation at 72°C for 1 min, and a final elongation step at 72°C for 10 min. Except for two strains identified as *Aspergillus niger* with the *ITS* markers, the other selected antagonistic fungi were further identified at the species level with *tef1-α* (translation elongation factor 1 alpha) used as marker. PCR amplification was conducted with the pairs of primers given in **Table 2**, and the same protocol as described above. For *Apiospora* species identification, two sequences were amplified in the large subunit ribosomal RNA (*LSU*) and beta-tubulin (*TUB*) genes with primers specified in **Table 2** (Tian et al., 2021; Kwon et al., 2022). The PCR protocol was the same as described above, except for the annealing temperature which was 56°C. For *Alternaria* and *Trichoderma* species identification, the RNA polymerase II second largest subunit gene (*rpb2*) was amplified using primers given in **Table 2**. The PCR protocol for *rpb2* was adapted from Liu et al. (1999) with an initial step at 95°C for 3 min, followed by 35 cycles at 95°C for 30 s, 55°C for 30 s, a ramp of 3 min that increased the temperature by 0.2°C per second from 55°C to 72°C, and a final step at 72°C for 10 min. PCR products were then sequenced by Eurofins Genomics (Europe Pharma and Diagnostics Products & Services Sanger/PCR GmbH, Konstanz, Germany). For genus-level classification, all sequences were analyzed with the R package dada2 (Callahan et al., 2016) and compared with representative sequences available in the UNITE database (Abarenkov et al., 2022). For species identification, the sequences were analyzed using the NCBI tool BLASTn of the GenBank database (www.ncbi.nlm.nih.gov/BLAST/; Johnson et al., 2008). All sequences were deposited in GenBank with assignment of their accession numbers (**Table 1**).

**Table 2:** PCR primers used for species identification of antagonistic *Apiospora*, *Alternaria* and *Trichoderma* strains isolated from wild Brassicaceae seeds.

DNA marker	Primer name	Primer sequence (5’-3’)	Direction	Species identification	References
*tef1-α*	EF1-728F	CATCGAGAAGTTCGAGAAGG	Forward	*Alternaria, Trichoderma* and *Apiospora*	(Carbone and Kohn, 1999; Hammad et al., 2021)
	EF1-986R	TACTTGAAGGAACCCTTACC	Reverse	*Alternaria*	(Carbone and Kohn, 1999)
	TEF1LLErev	AACTTGCAGGCAATGTGG	Reverse	*Trichoderma*	(Hammad et al., 2021)
	EF1-1567R	ACHGTRCCRATACCACCRATCTT	Reverse	*Apiospora*	(Rehner and Buckley, 2005)
*rpb2*	RPB2–5F2	GAYGAYMGWGATCAYTTYGG	Forward	*Alternaria* and *Trichoderma*	(Liu et al., 1999; Hammad et al., 2021)
	fRPB2–7cR	CCCATRGCTTGYTTRCCCAT	Reverse		(Liu et al., 1999)
*LSU*	LR0R	ACCCGCTGAACTTAAGC	Forward	*Apiospora*	Vilgalys unpublished
	LR5	TCCTGAGGGAAACTTCG	Reverse		(Vilgalys and Hester, 1990)
*TUB*	Bt2a	GGTAACCAAATCGGTGCTGCTTTC	Forward	*Apiospora*	(Glass and Donaldson, 1995; Tian et al., 2021)
	Bt2b	ACCCTCAGTGTAGTGACCCTTGGC	Reverse		(Glass and Donaldson, 1995; Tian et al., 2021)

### 2.4 *In vitro* sensitivity of *A. brassicicola* to antagonistic strains in liquid media using a nephelometric assay

The nine selected antagonistic strains which strongly inhibited the growth of *A. brassicicola* on PDA were tested for their antibiosis capacity in liquid media. Experiments were performed using 96-well plates and laser nephelometry as described in Joubert et al. (2010). Conidial suspensions of *A. brassicicola* were prepared from 7-day-old fungal colonies grown on PDA in the dark at 22°C. Protocol to obtain fungal filtrates from each antagonist was adapted from Hammad et al. (2021). Five mycelial explants of 5-mm diameter were collected at the periphery of 7-day-old fungal colonies grown on PDA medium in the dark at 22°C. Explants were then ground with 50 mL of autoclaved Potato Dextrose Broth (PDB, Grosseron) for 20 s with a SORVALL^®^ omni-mixer 17106 grinder. Five uninoculated PDA explants were similarly ground in 50 mL of PDB and served as negative control conditions. The resulting grindings were transferred into 250 mL sterile Erlenmeyer flasks and incubated at 22°C in the dark on a InforsHT shaker minitron with continuous shaking at 100 rpm for 7 days. To eliminate fungal residues, cultures were first filtered using a 1-mm mesh strainer and collected in a 50 mL sterile Falcon tube. To homogenize experimental conditions among antagonistic strains, the pH of filtrates was adjusted to 5.2 with HCl 1M or NaOH 1M. Cultures were sterilized through 0.2-µm filters from Dutscher (Bernolsheim, France). Subsequently, a nephelometric assay was conducted to measure the impact of fungal filtrates from each antagonist on the growth of *A. brassicicola*. Autoclaved PDB was inoculated with 10% (v/v) of a 10^5^ conidia/mL suspension of *A. brassicicola*, 10% (v/v) of sterile fungal filtrate and 1% (v/v) of solvent (DMSO or MeOH). Inoculated PDB supplemented with PDA explants without fungal cultures and DMSO or MeOH as solvent, served as positive control, and PDB medium supplemented with each fungal filtrate and DMSO or MeOH as solvent, as negative controls. For all tested antagonists, *A. brassicicola* mycelial growth was measured according to Joubert et al. (2010) with the following parameters: 600 µL of suspension mix from each experimental condition were evenly distributed into three wells (200 µL per well). The plates were incubated at 25°C for 100 h in a 635-nm laser nephelometer (NEPHELOstar Plus BMG Labtech, Offenburg, Germany) and *A. brassicicola* growth was recorded at 10-mn intervals, resulting in a total of 600 cycles. Prior to each measure, the plate underwent a double orbital shaking at 200 rpm. Laser conditions were optimized with a beam focus of 2.5 mm and an intensity set at 80%. For each condition, three independent experiments were performed. Inhibition of *A. brassicicola* by each culture filtrate was calculated using the area under the curve (AUC) as described in Calmes et al. (2015).

### 2.5 *In vitro* sensitivity of *A. brassicicola* and the antagonistic strains to plant defense compounds using a nephelometric assay

To measure the impact of defense compounds on mycelial growth of *A. brassicicola* and of the nine selected antagonistic strains, experiments were conducted in liquid media using 96-well plates and laser nephelometry as described above. The tested defense compounds were the phytoalexin camalexin and the three classes of GLS-breakdown compounds: allyl-ITC derived from aliphatic GLS, benzyl-ITC from aromatic GLS, and indole-3-carbinol (I3C) from indolic GLS. To define optimal experimental conditions, preliminary nephelometric assays were performed to determine the IC50 values on *A. brassicicola* growth for each defense compound. A 50% mycelium growth inhibition was recorded at the following concentrations: 51 µM camalexin, 636 µM allyl-ITC, 195 µM benzyl-ITC and 845 µM I3C. Subsequently, for each nephelometric assay, autoclaved PDB was inoculated with 10% (v/v) of a 10^5^ conidia/mL suspension of *A. brassicicola* or of each antagonistic fungus and 1% (v/v) of defense compound at the IC50 values estimated on *A. brassicicola*. ITC were purchased from Sigma-Aldrich (Darmstadt, Germany) and camalexin was available in our laboratory (Sellam et al., 2007b). Stock solutions were prepared at the desired concentrations using DMSO and MeOH as solvents for camalexin and ITC respectively (Sellam et al., 2007b). Positive control conditions were prepared with 89% (v/v) of PDB, 10% (v/v) of a 10^5^ conidia/mL suspension from *A. brassicicola* or one of the antagonistic strain, and 1% (v/v) of solvent (DMSO or MeOH). Negative control conditions were prepared with 99% (v/v) of PDB and 1% (v/v) of solvent (DMSO or MeOH). Fungal growth of *A. brassicicola* and of each antagonistic strain was measured as described above.

### 2.6 *In vitro* sensitivity of *A. brassicicola* to fungal filtrates amended with plant defense compounds using a nephelometric assay

Nephelometric assays were also conducted to measure the combined effects on *A. brassicicola* mycelial growth of fungal filtrates from the nine selected antagonistic strains amended with one of the plant defense compounds: camalexin, allyl-ITC, benzyl-ITC or I3C. As described previously, for each specific defense compound and fungal filtrate combination, the control conditions were as follows: either defense compounds were replaced by their respective solvent, or fungal filtrates were replaced by PDA filtrates without fungal cultures. Inhibition of *A. brassicicola* by each combination of defense compound × fungal filtrate was calculated as described above under the same parameters.

### 2.7 Statistical analyses

All analyses of the nephelometric data were performed on AUC values. As data did not meet the assumptions of the ANOVA, the non-parametric Kruskal-Wallis test with an alpha threshold of 0.05 was used, followed by a Wilcoxon rank sum posthoc test to divide the statistical groups. All figures were created with the ggplot2 package (Wickham, 2009) in R Statistical Software (v4.3.0; R Core Team, 2023).

For each tested condition, inhibition of *A. brassicicola* by each antagonist was calculated using the area under the curve (AUC) as described in Calmes et al. (2015). Furthermore, a percentage of growth inhibition by each fungal strain on solid and liquid media, amended or not with a defense compound, was calculated as described by N’Guyen et al. (2021). The following equation that compares each AUC of *A. brassicicola* in presence of fungal filtrates and defense compounds (9 replicates for each condition) with the AUC of the corresponding control conditions was used:


$$\text{I}=\frac{\bar{AUC_{\mathrm{Abra}+S}}-AUC_{\mathrm{Abra}+\text{ X}}}{\bar{AUC_{\text{Abra }+\text{ S}}}}$$

With I = The percentage of inhibition of X on *A. brassicicola* growth (Abra). X could be either a fungal filtrate or a defense compound or a combination of defense compound × fungal filtrate. The bar above the control condition “*AUC_Abra + S_*” indicates the average of AUC (n = 9) of *A. brassicicola* (Abra) growth in presence of a solvent (S) which was DMSO for camalexin and MeOH for GLS.

Similarly, the following equation was used to calculate the impact of a defense compound on the growth of each antagonistic fungus:


$$\text{I’}=\frac{\bar{AUC_{AF+S}}-AUC_{AF+C}}{\bar{AUC_{AF+S\;}}}$$

With I’= The percentage of inhibition of a defense compound (C) on an antagonistic fungus (AF) growth.

In order to measure the potential synergistic effects between fungal filtrates and defense compounds on the growth of *A. brassicicola*, the Colby’s ratio, also known as Bliss method (Colby, 1967; Duarte and Vale, 2022) was simplified and used with the AUC values. A ratio between observed inhibition in the presence of both defense compounds and fungal filtrates as numerator, and a theoretical additive inhibition as denominator with fungal filtrates alone and defense compounds alone, was calculated as:


$$\text{Colby}’s=\;\frac{\bar{AUC_{Abra+S}}-\;AUC_{Abra+F+C}}{\bar{AUC_{Abra+S}}-\;AUC_{Abra+S+F}\times \left(1-x\right)}$$

With *AUC_Abra+F+C_* = The AUC of *A. brassicicola* growth in the presence of fungal filtrates (F) amended with defense compounds (C), and AUC_Abra+S+F_ = The AUC of *A. brassicicola* growth in the presence of fungal filtrates (F) amended with solvents (S). This condition is called “fungal filtrates alone”.

*x* was a fixed value of the inhibition percentage of a defense compound alone on *A. brassicicola*. Theoretically, *x* was equal to 0.5 since the IC50 was used for each compound. However, a Wilcoxon test compared observed values of inhibition percentages with the theoretical value 0.5. For each defense compound, in case of a significant difference between the observed inhibition and 0.5, the value of *x* was defined as the average of the growth inhibition of *A. brassicicola* by that defense compound. As a result, for camalexin and I3C, *x* = 0.5, for allyl-ITC, *x* = 0.35, and for benzyl-ITC, *x* = 0.45.

A Colby’s ratio > 1 indicates a synergistic effect between fungal filtrates and defense compounds which means that in presence of both fungal filtrates (F) and defense compounds (C), inhibition of *A. brassicicola* is higher than when there is addition of the inhibition percentages in presence of fungal filtrates alone and defense compounds alone (F+C together > F alone + C alone). When the ratio was equal to 1, there is an additive effect (F+C together = F alone + C alone). Finally, a ratio < 1 indicates an antagonistic effect (F+C together < F alone + C alone with F+C together > F alone and F+C together > C alone). In order to statistically differentiate between these effects, a Wilcoxon test was applied to compare the numerator (representing the observed effect of F+C) and the denominator (representing the theoretical additive effect of F+C) of the Colby’s ratio.

## 3 Results

### 3.1 Isolation and selection of antagonistic isolates on solid agar medium

From the seeds collected in different wild Brassicaceae plant populations, a total of 319 strains were isolated on the two solid media. Among them, based on optical microscopy observations and *ITS* sequences, 186 strains were identified as *Botrytis*, *Penicillium* or *Cladosporium* spp. (Lerenard et al., submitted), which are mostly described as opportunistic pathogens or weak antagonists (Oliver et al., 2001; Sarosh et al., 2009; Wang et al., 2018; Hassan et al., 2019). These isolates were therefore not tested against *A. brassicicola*. By dual culture assays on solid PDA medium, the remaining 133 isolates were screened to quantify their antagonistic activity against *A. brassicicola*. A great number of strains (N = 84) demonstrated some capacity to reduce the growth of *A. brassicicola* compared to the control, with percent inhibition values ranging from 19.3% (± 3.4) to 61.7% (± 1.3; Lerenard et al., submitted). For each host plant and independently of sampling sites, the three sporulating strains with the highest percent inhibition were selected (**Table 1**). Following PCR amplifications and sequence analyses, these nine antagonistic strains were identified at the species level (**Table 1** with the accession numbers). Four of those nine strains isolated from the three wild Brassicaceae species, belonged to the genus *Alternaria* (At 289, At 416, Cb 324 and Dv 245). Two species of *Apiospora* were identified on seeds collected from *A. thaliana* (At 199) and *C. bursa-pastoris* (Cb 322). One *Trichoderma viridarium* strain was isolated from *C. bursa-pastoris* (Cb 087) and two *Aspergillus niger* strains (Dv 028 and Dv 101) from *D. verna*.

### 3.2 *In vitro* antagonistic activity of the selected strains in liquid media

**Figure 1:**
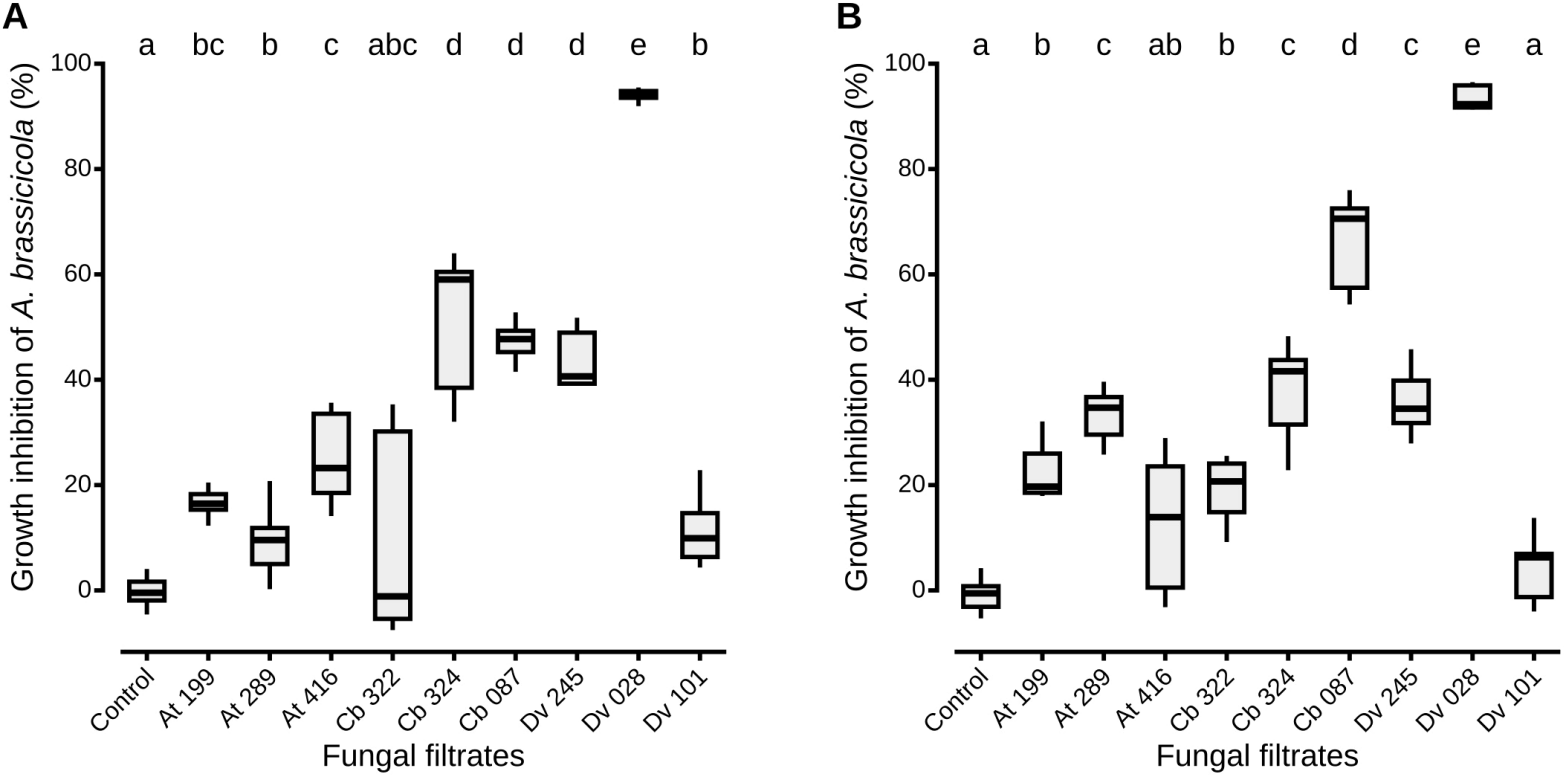
*A. brassicicola* growth inhibition *in vitro* by the nine most antagonistic strains isolated from wild Brassicaceae seeds. For each nephelometric assay, liquid PDB was inoculated with a 10^5^ conidia/mL suspension of *A. brassicicola* and fungal culture filtrates from each antagonist in either DMSO **(A)** or MeOH **(B)** as solvent. Control conditions correspond to filtrates obtained from cultures with uninoculated PDA explants. Boxes and horizontal lines represent the first and the third quartiles and medians respectively. Groups with the same letter are considered to be non-significantly different according to Wilcoxon rank-sum test (p < 0.05) with n = 9 (three independent experiments with three well replicates) for each condition.

The growth of *A. brassicicola* in liquid medium was measured by nephelometry in the presence of filtrates from each of the nine strains selected for their high inhibitory activity on PDA (**Figure 1**). Similar results as those described on PDA with live fungi were observed, as fungal culture filtrates from the different strains also reduced significantly the mycelial growth of *A. brassicicola*. Regardless of the solvent, percentages of mycelial growth inhibition ranged from 4.4 ± 6.1% to 93.6 ± 2.3% (**Figure 1**), and values obtained in the two solvents were highly corelated (Pearson correlation coefficient, r = 0.94, p = 0.0016). However, distinct patterns among strains were observed when their antagonistic impact on solid (PDA) and in liquid (PDB) media were compared: while two *Alternaria* strains (Cb 324 and Dv 245) and *T. viridarium* (Cb 087) exhibited similar inhibitory activity in both media, others displayed considerable differences (**Table 1**; **Figure 1**). For example, the filtrate from one *Aspergillus niger* strain (Dv 028) demonstrated the highest inhibition percentage in liquid media (**Figure 1**), with an increase of 124% of its inhibitory effect compared to what was observed on PDA (**Table 1**). On the other hand, four fungal culture filtrates from two *Apiospora* (At 199 and Cb 322), one *Alternaria* (At 416), and one *Aspergillus* (Dv 101) did not have a significant inhibitory effect on *A. brassicicola* in the MeOH solvent (**Figure 1**). However, they all significantly reduced the growth of *A. brassicicola* on PDA with percentages of inhibition ranging from 39.4 ± 3.7 to 51.9 ± 1.6% for At 199, At 416 and Dv 101, and up to 61.7 ± 3.2% for the Cb 322 isolate (**Table 1**). When the origin of the antagonistic strains is considered, strains isolated from seeds of *C. bursa-pastoris* or *D. verna* exhibited contrasted inhibition percentages in solid (PDA) and liquid (PDB) media while all strains isolated from seeds of *A. thaliana* had a very similar impact on *A. brassicicola* mycelial growth (**Table 1**; **Figure 1**). This variability in inhibition was also present when strains that belong to the same fungal genus were compared. For example, among *Aspergillus niger* strains, filtrate from Dv 028 exhibited the highest inhibition percentage and from Dv 101 the lowest, in both DMSO and MeOH solvents. By contrast, for the two *Apiospora* strains (At 199 and Cb 322), inhibition of *A. brassicicola* was much more pronounced on PDA than in liquid media in both solvents (**Table 1**; **Figure 1**).

### 3.3 *In vitro* sensitivity of *A. brassicicola* and the antagonistic strains to plant defense compounds using a nephelometric assay

**Figure 2:**
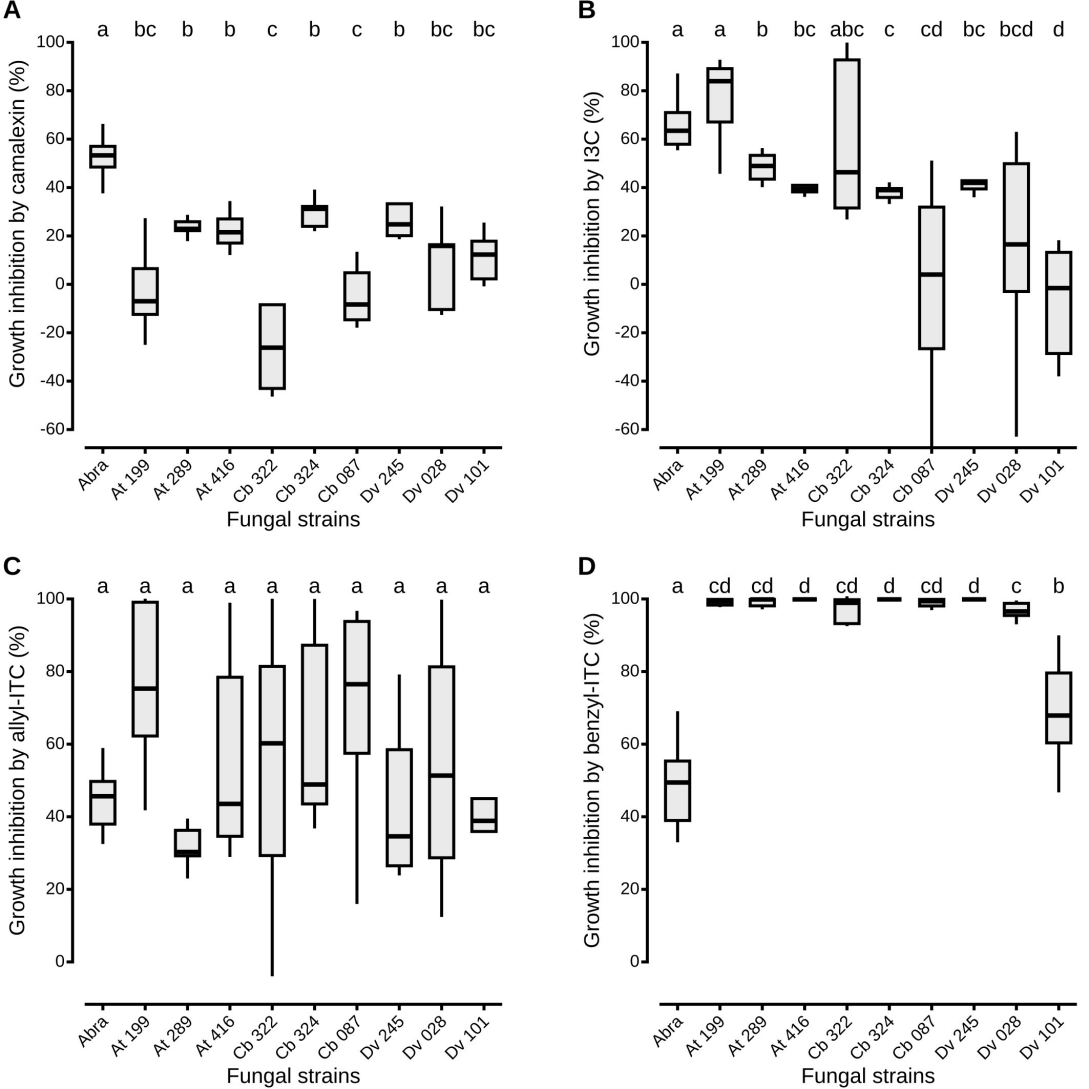
Antagonistic fungi growth inhibition *in vitro* by plant defense compounds. The liquid media PDB was amended with camalexin **(A)**, I3C **(B)**, allyl-ITC **(C)** or benzyl-ITC **(D)**, prepared at IC50 determined on *A. brassicicola* growth (see Material and Methods section). Negative percentages of inhibition mean that fungal growth in presence of the defense compound was higher than its growth without the compound. Abra means *A. brassicicola*. Isolate references are given in **Table 1**. Boxes and horizontal lines represent the first and the third quartiles and medians respectively. Groups with the same letter are considered to be non-significantly different according to Wilcoxon rank-sum test results (p < 0.05) with n = 9 (three independent experiments with three well replicates) for each condition.

To assess the effects of defense compounds on the growth of antagonistic fungi, mycelial growth of each strain was measured in liquid media amended with camalexin, allyl-ITC, benzyl-ITC or I3C. For each assay, concentrations of the defense compounds corresponded to the IC50 values determined on *A. brassicicola* growth (**Figure 2**). All antagonistic strains were significantly less inhibited by camalexin than *A. brassicicola*, with percentages of inhibition that ranged from -28.0% (± 16.6) to 29.7% (± 6.1; **Figure 2A**). Two strains, one *Apiospora* (Cb 322) and *Trichoderma viridarium* (Cb 087), had a percentage of inhibition lower than 0% which indicates that fungal growth in presence of camalexin was higher than without the compound. Similarly, except for one *Apiospora* isolate (At 199), the growth of all the other strains was significantly less impacted by I3C than *A. brassicicola* with a gradient in inhibition percentages that ranged from -7.0% (± 22.4) to 49.0% (± 6.2; **Figure 2B**). By contrast, all antagonistic strains were as inhibited as *A. brassicicola* in the presence of allyl-ITC with no significant differences among the different isolates (**Figure 2C**). Finally, all antagonistic strains were significantly more inhibited than *A. brassicicola* in the presence of benzyl-ITC. Only one isolate of *Aspergillus niger* (Dv 101) was significantly less inhibited than all the other antagonistic strains but still significantly more than *A. brassicicola* (**Figure 2D**).

### 3.4 *In vitro* sensitivity of *A. brassicicola* to fungal filtrates amended with plant defense compounds using a nephelometric assay

**Figure 3:**
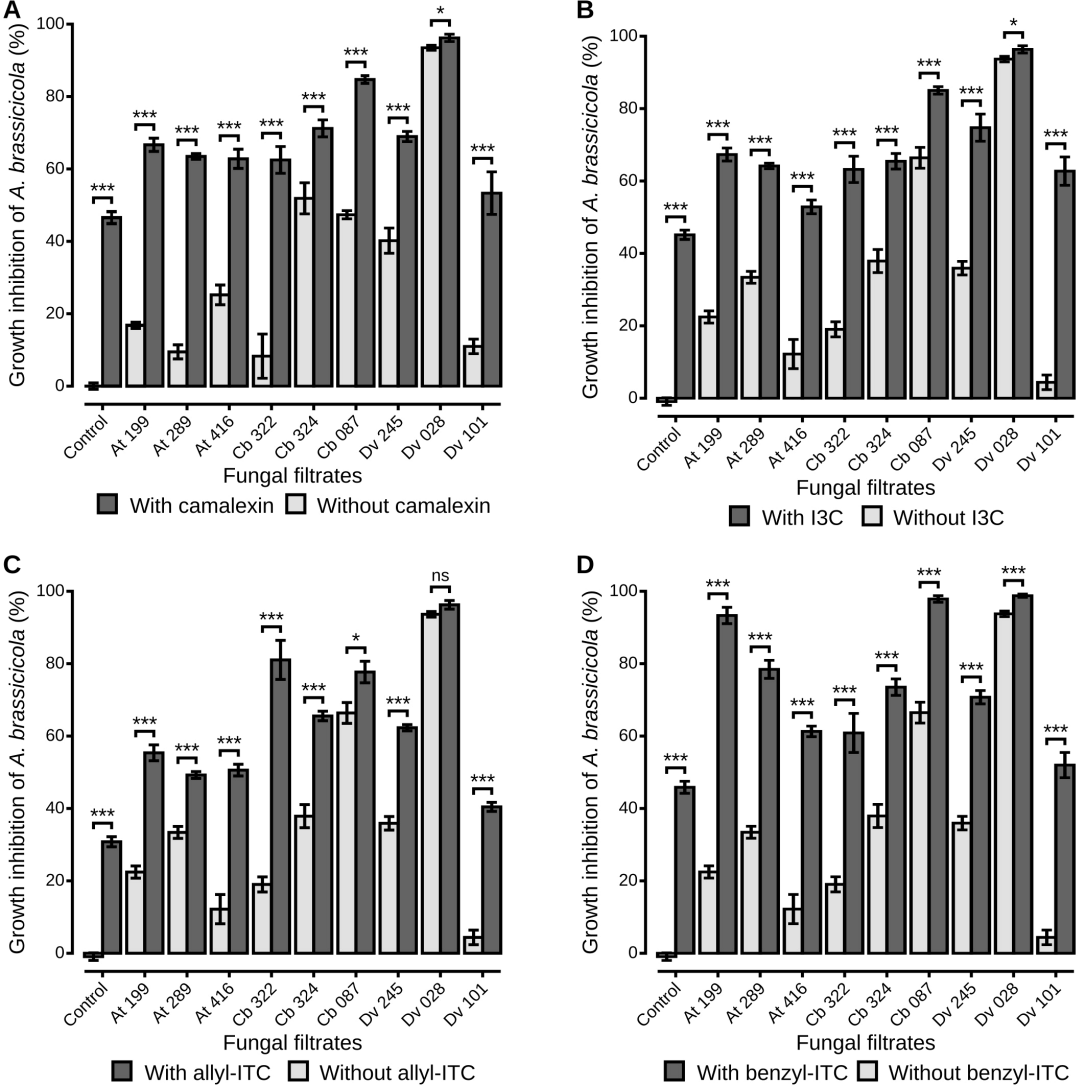
*A. brassicicola* growth inhibition *in vitro* by fungal culture filtrates amended or not with plant defense compounds. The liquid media PDB was amended with fungal filtrates without defense compounds (light grey bars), or amended with fungal filtrates and plant defense compounds (dark grey bars). Plant defense compounds were camalexin **(A)**, I3C **(B)**, allyl-ITC **(C)** or benzyl-ITC **(D)**, prepared at IC50 determined on *A. brassicicola* growth (see Material and Methods section). Control condition corresponds to *A. brassicicola* growth with culture filtrates from uninoculated PDA explants. Barplots represent the mean ± standard error. Stars indicate significant differences between each culture filtrate with and without defense compounds, according to a Wilcoxon rank-sum test (p > 0.05 non significant, p < 0.05 single star, p < 0.01 double star, p < 0.001 triple star) with n = 9 (three independent experiments with three well replicates) for each condition.

The combined effects of fungal filtrates and defense compounds on the growth of *A. brassicicola* were measured for the nine selected strains and the four classes of defense compounds (**Figure 3**). For each combination, fungal culture filtrates from the nine strains reduced significantly the mycelial growth of the pathogen in presence of camalexin (**Figure 3A**) and allyl-ITC (**Figure 3C**;Wilcoxon sign-rank test, p < 0.05 for all comparisons). The same result was observed in presence of I3C (**Figure 3B**), except for one *Alternaria* (At 416) filtrate which did not have a significant negative impact on *A. brassicicola*. Similarly, all filtrates reduced significantly the mycelial growth of the pathogen in presence of benzyl-ITC (**Figure 3D**), except for one *Aspergillus* (Dv 101). Moreover, significant differences among strains were also observed in presence of the different defense compounds (Wilcoxon sign-rank test, p < 0.05 for all comparisons). In particular, when the fungal filtrates were amended with camalexin, I3C, and allyl-ITC, *T. viridarium* (Cb 087) and *Aspergillus niger* (Dv 028) culture filtrates demonstrated the highest inhibition percentages on *A. brassicicola* compared to all other strains (**Figures 3A–C**). In presence of benzyl-ITC, filtrates from these two strains as well as from one *Apiospora* (At 199) reduced significantly the growth of *A. brassicicola* more than any other strain (**Figure 3D**). The percentage of inhibition of *A. brassicicola* was also significantly higher when filtrates were amended with defense compounds than without (**Figure 3**). Only the culture filtrate from on *Aspergillus niger* (Dv 028) amended with allyl-ITC did not show a significantly higher inhibition than filtrate alone, which was expected as the culture filtrate alone exhibited an inhibition percentage close to 100% (**Figure 3C**).

**Figure 4:**
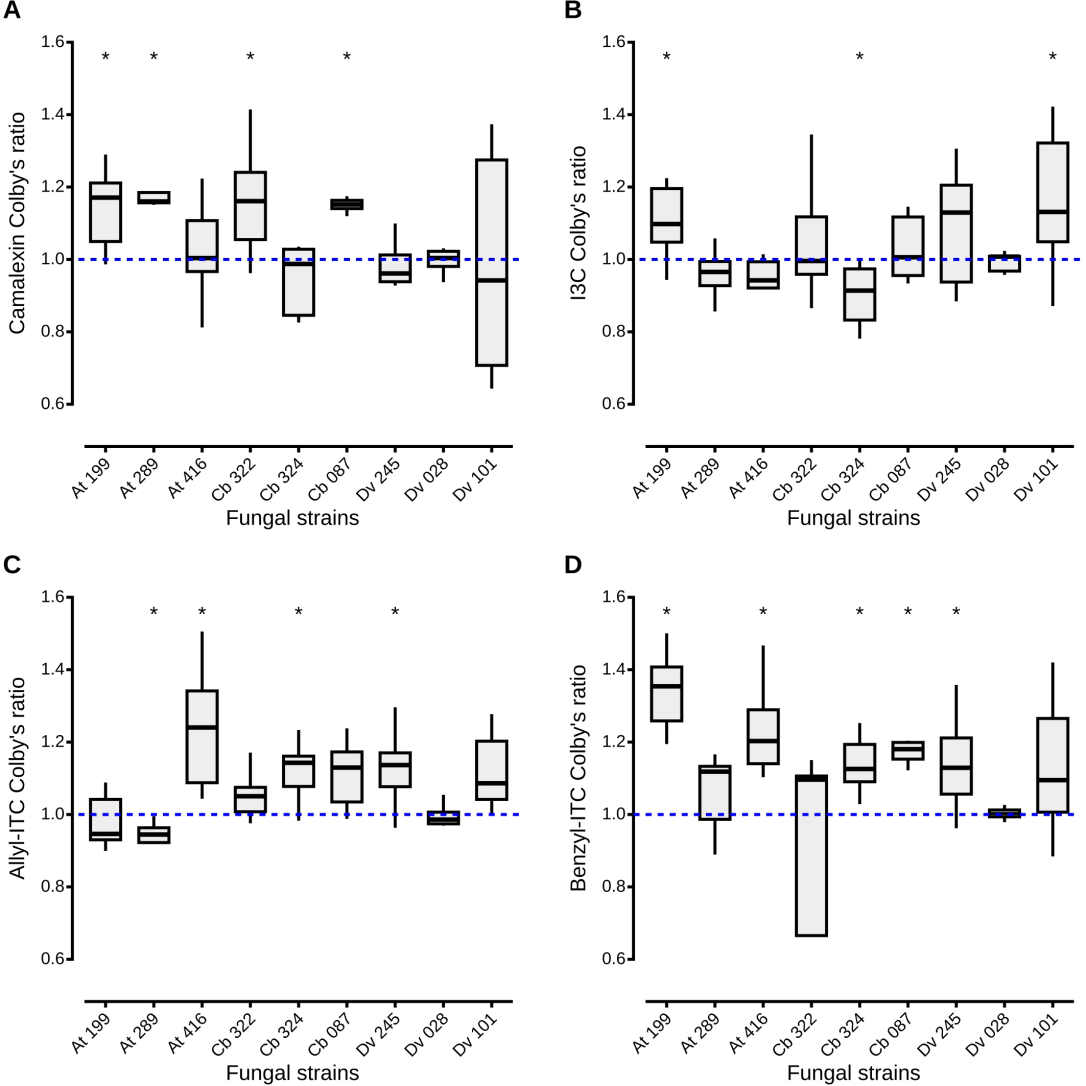
Synergistic, additive and antagonistic effects between fungal filtrates and plant defense compounds on *A. brassicicola* growth. Colby’s method, also known as Bliss method, was adapted to calculate a ratio between observed and theoretical inhibition of *A. brassicicola* growth by fungal culture filtrates amended or not with plant defense compounds. A Colby’s ratio > 1 indicates a synergistic effect between fungal filtrates and defense compounds, a ratio < 1, an antagonistic effect. When the ratio is equal to 1, there is an additive effect. Boxes and horizontal lines represent the first and the third quartiles and medians respectively. Stars indicate a significant difference between observed inhibition and theoretical inhibition of *A. brassicicola* using the Wilcoxon rank-sum test ( p < 0.05) with n = 9 for each condition.

As inhibition of *A. brassicicola* by the fungal culture filtrates seemed reinforced in the presence of defense compounds, the Colby’s ratio (Colby, 1967) was calculated to reveal potential synergistic effects of the different combinations of fungal filtrates from the nine antagonistic strains and the four classes of defense compounds. A Colby’s ratio > 1 indicated synergistic effects between camalexin and fungal filtrates from four antagonistic strains, two *Apiospora* (At 199 and Cb 322), one *Trichoderma* (Cb 087) and one *Alternaria* (At 289), isolated from host plants that synthesize that compound (**Figure 4A**). For other antagonistic strains, their effects on *A. brassicicola* in presence of camalexin were additive: the inhibition percentages were not higher than expected from the added inhibition observed in presence of the fungal filtrate alone and camalexin alone. For the other defense compounds, Colby’s ratios > 1 indicated synergistic effects between two, three and five strains and I3C (**Figure 4B**), allyl-ITC (**Figure 4C**), and benzyl-ITC (**Figure 4D**) respectively. Finally, for two *Alternaria* strains (Cb 324 and At 289), the inhibition percentages were significantly lower than expected from the added inhibition observed in presence of the fungal filtrate alone and I3C alone or allyl-ITC alone respectively, as Colby’s ratio was < 1 (**Figures 4B, C**). On the other hand, culture filtrates from five antagonistic strains (At 199, At 416, Cb 324, Cb 087, Dv 245), isolated from the three host plants, had a negative impact on *A. brassicicola* reinforced in presence of benzyl-ITC (**Figure 4D**). To summarize, except for the filtrates from one *Aspergillus* strain Dv 028 which showed only additive effects irrespective of the defense compound, synergistic effects were observed for all the other fungal culture filtrates in presence of at least one of the four classes of defense compounds.

## 4 Discussion

Exploration of the microbiota associated with wild plant species has rarely been conducted while it might harbor microorganisms which could be used as potential BCA (Luu et al., 2021). To identify potential antagonists to the seed-borne pathogen *A. brassicicola*, we collected seeds in populations of *A. thaliana, C. bursa-pastoris* and *D. verna* and isolated a large number of fungal strains which significantly reduced the growth of *A. brassicicola* (N = 84 out of 319 strains). The three most antagonistic strains isolated on each of the three sampled plant species belonged to *Alternaria, Apiospora, Trichoderma* and *Aspergillus*. Variability in inhibitory effect against *A. brassicicola* seemed independent of fungal genus and origin of the strain (host plant species and sampling site). However, distinct patterns among strains were observed when the inhibitory effects on *A. brassicicola* were compared in solid and liquid media as previously documented for other potential BCA (Yassin et al., 2021; Yi et al., 2022). As all four fungal genera are known to produce anti-fungal compounds (Feng and Ma, 2010; Hermosa et al., 2014; Idan et al., 2017; Overgaard et al., 2023), differences in inhibition might be due to the underlying mechanisms that explain the antagonistic potential of the isolated strains in dual culture assays and culture filtrates. *In vitro*, direct inhibition of plant pathogens may involve antibiosis through production of toxic molecules, competition for nutrients or space, or mycoparasitism. For example, for one filtrate from an *Aspergillus niger* isolate (Dv 028), in nephelometric assays, toxic compounds present in the culture filtrates might have quickly inhibited the growth of *A. brassicicola*, whereas on solid medium, their diffusion and impact on the growth of the pathogen might have required several days. On the other hand, on PDA and in presence of fungal colonies, antagonism might be due to competition for resources as observed for two strains of *Apiospora* (Cb 322 and At 199). Their rapid growth on the plates (personal observation) might have given them a competitive advantage over *A. brassicicola*, without production of toxic compounds as confirmed by physical contacts between the fungal colonies and weak inhibition in the nephelometric assays (**Figure 1**). Finally, for two *Alternaria* (At 289 and At 416) and one *Aspergillus niger* (Dv 101) culture filtrates characterized by weak inhibition in nephelometric assays, high inhibition percentage with spatial separation between fungal colonies in the dual culture assays indicated potential antibiosis (Zhao et al., 2020). Solid metabolites and/or volatile organic compounds with antifungal properties against *A. brassicicola* might not be soluble in aqueous medium such as PDB (Ali et al., 2015). As a consequence, even though these metabolites are not identified, differences in inhibitory effect on *A. brassicicola* between solid and liquid media for some of the strains might be due to the characteristics of the antifungal molecules produced either constitutively in all assays, or after induction by the pathogen in the dual culture assays as observed in other studies (Nonaka et al., 2015). Moreover, these findings confirm our hypothesis that the seed compartment of wild Brassicaceae harbors antagonistic fungi which might contribute to the resistance against *A. brassicicola* reported in some species (Cooke et al., 1997; Westman et al., 1999).

If resistant wild Brassicaceae species host antagonistic fungi with an inhibitory effect on *A. brassicicola*, the seed microbiota must be able to cope with high levels of constitutive or induced plant defenses. To our knowledge, no study has investigated the sensitivity to plant secondary metabolites of non-pathogenic strains compared to pathogens. Regarding *A. brassicicola*, inhibitory effects of the phytoalexin camalexin (Sellam et al., 2007b; Tao et al., 2022), and of the main three classes of GLS-derived ITC: allyl-ITC, benzyl-ITC (Sellam et al., 2007b; Calmes et al., 2015) and I3C (Pedras and Hossain, 2011) have been demonstrated *in vitro*, with differences in sensibility of *A. brassicicola* to the different classes of defense compounds. For example, mycelial growth of *A. brassicicola* strain Abra43 was more impacted by camalexin compared to benzyl and allyl-ITC in a study conducted by Sellam et al. (2007b), and confirmed by our IC50 values calculated for the nephelometric assays (see Materials and methods section). Furthermore, *A. brassicicola* was more strongly impacted by aliphatic GLS and camalexin than by indolic GLS when inoculated to *A. thaliana* mutants with different defense profiles (Buxdorf et al., 2013). Differences in sensibility might be explained by more or less efficient detoxification of the plant defense compounds. For example, detoxification of camalexin by fungal pathogens such as *A. brassicicola, Sclerotinia sclerotiorum, Rhizoctonia solani* and *Botrytis cinerea* has been documented and occurs at different rates (Pedras and Abdoli, 2013). Similarly, in a recent review, Plaszkó et al. (2021) compiled extensive data on detoxification of GLS-derived ITC mostly by pathogenic fungi and non-pathogenic mycorrhizal fungi, Brassicaceae and non-Brassicaceae specialists, and describes a wide range of specific and less specific detoxification processes. Differences in sensitivity between pathogens and non-pathogenic fungal strains are expected.

Our results highlight the lower impact of the indolic phytoalexin camalexin and the GLS-derived indolic I3C on the nine antagonistic strains than on *A. brassicicola*. The *Trichoderma viridarium* strain (Cb 087) and both *Apiospora* (At 199 and Cb 322) had even a negative inhibition percentage in presence of camalexin which means that they were stimulated by the compound. Tolerance as expressed by the low impact of camalexin on the growth of all isolates might be due to efficient detoxification, maybe partly explained by the capacity of *Trichoderma, Alternaria* and *Aspergillus* to induce camalexin production (Thomma, 2003; Salas-Marina, 2011; Velázquez-Robledo et al., 2011; Contreras-Cornejo et al., 2014). Antagonistic fungi were also significantly less impacted by I3C than *A. brassicicola* but there is little data on potential mechanisms (Plaszkö et al., 2021). Pedras and Hossain (2011) did report that indolic GLS were not metabolized by *A. brassicicola*, although low myrosinase activity was detected in the mycelia. Furthermore, the generalist pathogens *S. sclerotiorum* and *R. solani* could metabolize the corresponding desulfo-GLS, but not *A. brassicicola* (Pedras and Hossain, 2011). Antagonistic strains isolated in our study might therefore be more tolerant to I3C than *A. brassicicola* but there is no data to support this hypothesis.

Regarding the other classes of GLS-derived ITC, seed-associated fungi were as inhibited as *A. brassicicola* in the presence of allyl-ITC. Direct impacts of aliphatic derived ITC have been extensively documented for a large number of fungi (Plaszkó et al., 2021). Allyl-ITC seem to primarily act as oxidative stress agents that can be detoxified, thanks to specific mechanisms, by many strains that “live in close contact with Brassicaceae” including *A. brassicicola*. Other unexplored mechanisms like the production of efflux pumps or cell-wall repairs might also explain the tolerance to allyl-ITC (Sellam et al., 2007a; Plaszkó et al., 2021). *Alternaria brassicicola* and antagonistic strains might therefore share similar mechanisms to cope with this group of GLS-derived compounds. Unexpectedly, it is also reported that allyl-ITC can be used by fungi as source of nutrients. For example, the majority of fungal endophytes isolated from roots of horseradish *Armoracia rusticana* could metabolize aliphatic GLS quite efficiently, and use sinigrin, an aliphatic GLS decomposed into allyl-ITC, as source of carbon (Szűcs et al., 2018).

Finally, seed-associated fungi were more inhibited than *A. brassicicola* in presence of benzyl-ITC derived from aromatic GLS. Benzyl GLS and benzyl-ITC are efficiently metabolized by *A. brassicicola*, and not by *S. sclerotiorum* and *R. solani* (Pedras et al., 2021). This result suggests that *A. brassicicola* has developed mechanisms to detoxify more efficiently benzyl-ITC than other fungi, like maybe the nine antagonistic strains isolated in this study from wild Brassicaceae. By contrast, the majority of the root endophytes isolated by Szűcs et al. (2018) were not inhibited by aromatic GLS encountered in the host plant as most of them could decompose this class of GLS as efficiently as aliphatic GLS. Even though host plant characteristics and identity of the isolated strains might explain some of the differences between studies, contrasted results emphasize the lack of data on the impacts of plant defense compounds on the associated microbiota and highlight the need for further investigation on the underlying mechanisms.

Even though the mechanisms underlying inhibition of *A. brassicicola* by seed-associated fungi are not fully described, our data indicate that all defense compounds could enhance the antagonistic effects of the nine isolated strains on the pathogen. To our knowledge, no study had explored previously the combination of fungal endophytes and plant defenses on suppression of pathogen growth. Reinforcement of the antagonistic potential of the isolated strains might be due to different mechanisms. In our study, for one *T. viridarium* (Cb 087), two *Apiospora* (At 199 and Cb 322) and one *Alternaria* (At 289) strains, combination of the fungal filtrates and the phytoalexin camalexin exhibited synergistic effects according to Colby’s ratio (Colby, 1967). The fact that all corresponding strains were isolated from *A. thaliana* and *C. bursa-pastoris* which produce camalexin (Thomma et al., 1999; Jimenez et al., 1997), support the hypothesis that these strains may be adapted to their host plant and, in presence of camalexin, their toxicity on *A. brassicicola* may be reinforced. However, underlying mechanisms remain unknown and have rarely been illustrated. One example is given by Saravanakumar et al. (2016) who reported enhanced antagonistic activity on *Fusarium* wilt disease due to synergy of antimicrobial hydrolytic enzymes and metabolites produced by *Trichoderma* strains. More generally, very few studies to date focus on the synergistic impact of ITC and non-ITC compounds on plant pathogens (Plaszkó et al., 2021). For the remaining antagonistic strains, according to Colby’s ratio (Colby, 1967), either additive or antagonistic effects between fungal filtrates and the other plant defense compounds were observed. Additive effects were the most common and probably the most expected as filtrates amended with defense compounds act like a combination of two different inhibitors on *A. brassicicola*. In pharmacology studies, the combination of drugs with the same target, are expected to have additive effects (Wagner and Ulrich-Merzenich, 2009). In our case, defense compounds and some fungal metabolites can have similar targets. For example, camalexin and ITC target cell walls of fungi (Sellam et al., 2007a; Pereira et al., 2020). *Aspergillus, Alternaria* and *Trichoderma* are also able to produce cell wall degrading enzymes (CWDE) such as chitinases, proteases and β-glucanase which target cell walls and membranes of fungi (Sharaf, 2005; Brzezinska and Jankiewicz, 2012; Rehman et al., 2014; Rajesh et al., 2016; Dutta et al., 2022). Antagonistic effects between fungal filtrates and defense compounds were observed only for two combinations with filtrates from two *Alternaria arborescens* strains, Cb 324 and At 289, in presence of I3C and allyl-ITC respectively. Antagonism might be due to limitation or suppression of the effect of one of the inhibitors by the other (Kenakin, 2017). However, as for previous observations, understanding how the combination of plant defense compounds and molecules produced by fungal antagonists impacts the growth of *A. brassicicola* requires further studies.

## 5 Conclusion

Future research should get insights into the background mechanisms that explain the antagonistic potential of the fungal strains isolated from the seed microbiota. How fungal endophytes are able to inhibit the growth of a necrotrophic seed-borne plant pathogen requires a better understanding of the interactions between the pathogen and the endophytes, but also between the host plant and the endophytes. Antagonistic strains must cope with the defense compounds of the host plants. Further insights into detoxification of toxic compounds and possible other mechanisms are needed. Moreover, the observed potential synergistic effects between defense compounds and fungal filtrates emphasize the need for further studies to elucidate the molecular bases of the interactions. A better understanding of the interactions between host plants, pathogens and fungal endophytes is also needed to develop sustainable biocontrol strategies.

## Data Availability

The datasets presented in this study can be found in online repositories. The names of the repository/repositories and accession number(s) can be found in the article.
